# Changes in selected metabolic parameters in patients over 65 receiving hydrochlorothiazide plus amiloride, atenolol or placebo in the MRC elderly trial

**DOI:** 10.1186/s12872-016-0368-2

**Published:** 2016-10-04

**Authors:** Damian J. Damian, Roseanne McNamee, Matthew Carr

**Affiliations:** 1 Community Health Department, Kilimanjaro Christian Medical Centre, P. O. Box 3010, Moshi, Tanzania; 2 Biostatistics, Institute for Population Health, University of Manchester, Manchester, UK

**Keywords:** Anti-hypertensive, Hydrochlorothiazide, Amiloride, Atenolol, Metabolic parameters, MRC

## Abstract

**Background:**

Treatment of hypertension reduces incidence of stroke, myocardial infarction and heart failure perhaps partly by controlling different metabolic parameters. There is limited information regarding the changes in potassium, sodium, weight, cholesterol and glucose levels in patients using anti-hypertensives. This study aimed to determine changes in potassium, sodium, glucose, cholesterol, weight, urea and urate levels in patients using anti-hypertensives. Furthermore, to describe these changes and differences between the atenolol, hydrochlorothiazide plus amiloride and placebo arms of the Medical Research Council (MRC) elderly randomised controlled trial.

**Methods:**

Patients were randomly allocated to one of the three treatment arms. Measurements were taken at baseline, end of year one and end of year two in 4396 subjects. Linear Mixed Models (LMM) were used to determine the longitudinal profiles of sodium, potassium, weight, cholesterol, glucose, urea and urate. Estimates of changes within groups and difference between groups were obtained.

**Results:**

Patients randomised to receive hydrochlorothiazide + amiloride experienced a significantly greater mean reduction in potassium, sodium and weight compared to placebo at end of year one - mean differences in change −0.18 mmol/L, (95 % CI: −0.21, −0.15); −1.45 mmol/L, (95 % CI: −1.62, −1.29) and −0.46 kgs (95 % CI: −0.73, −0.20) respectively, and greater increases in cholesterol, urea and urate - mean differences in change 0.16 mmol/L, (95 % CI: 0.10,0.22); 0.77 mmol/L, (95 % CI: 0.68, 0.87) and 53.10 μmol/L, (95 % CI: 49.35, 56.85) respectively. Changes were in the same direction but smaller in the atenololarm except for potassium and weight (increases). No group differences in glucose were found.

**Conclusion:**

Results were in line with expectation except for lack of change in glucose in the hydrochlorothiazide + amiloride arms.

**Electronic supplementary material:**

The online version of this article (doi:10.1186/s12872-016-0368-2) contains supplementary material, which is available to authorized users.

## Background

Hypertension is present in at least 25 % of adults under the age of 50, and 50 % of those who are older [1]. It is responsible for 13 % of global deaths, 55 % of cerebrovascular disease, 45 % of heart disease and 61 % of cardiovascular deaths, and further linked to incidences of kidney disease, affecting high, low and middle-income countries [2]. Those living with uncontrolled hypertension grew from 605 million to 978 million worldwide between 1980 and 2008, making it highly relevant to contemporary medical practice [3].

Treatments for hypertension - which aim to reach and maintain acceptable blood pressure levels (<140/90 mmHg) - include angiotensin converting enzyme inhibitors (ACEs), angiotensin receptor blockers (ARBs), calcium channel blockers (CCBs), diuretics and beta-blockers. In the UK, recommended first line treatments have changed over time; CCBs are currently favoured as first line treatment for those aged over 55 years and for some ethnic groups at all ages, but thiazide or thiazide-like diuretics may also be recommended as first- or second-line therapy in certain cases [1, 4]. In the US, the recommendations for first line treatments in the 2014 - 8^th^Joint National Committee on Prevention, Detection, Evaluation, and Treatment of High Blood Pressure (JNC) report continue to include thiazide type diuretics as well as ACEs, ARBs and CCBs [5]. Diuretics were found to be the most commonly prescribed drug in 2009–2010 in the US National Health and Nutrition Examination Survey population with the most common type being thiazide type diuretics [6, 7]. A thiazide diuretic, hydrochlorothiazide + amiloride, was used in the UK MRC trial which we report on in this paper; this drug remains in common use at the time of writing [8].

Thiazide diuretics lower blood pressure by blocking reabsorption of sodium ions in the distal renal convoluted tubule and therefore draw water to be excreted as urine. This lowers blood volume leading to a reduction in cardiac output, and a reduction in peripheral resistance [1, 9]. Increased excretion of sodium, and perhaps some weight loss, are expected [10, 11]. However, their use is also associated with increased excretion of potassium- in extreme cases causing hypokalaemia - due to an increased exchange rate in potassium for sodium [12–16]. To counteract this, the use of a potassium sparing diuretic – on their own only weakly diuretic – may be used in combination.

Thiazide diuretics are known to increase serum urate concentrations, potentially increasing the occurrence of gout and gouty attacks and may also lead to low concentrations of urea [8, 17–19]. Moreover, the use of thiazide diuretics - and beta-blockers - is known to affect glucose homeostasis through potential *β-pancreatic* cell damage; they are associated with undesirable metabolic changes including decreased insulin secretion and sensitivity and decreased glucose tolerance [20, 21]. By causing increased excretion of potassium, thiazides can worsen glucose intolerance as low potassium inhibits insulin secretion [22], although there is limited evidence to support this [23]. However, this leads to an aggravated metabolic profile and an increased risk of new-onset diabetes mellitus [21, 24].

The 1980s UK MRC hypertensive trial among patients aged over 65 compared three treatment regimens: hydrochlorothiazide combined with potassium sparing amiloride, the beta blocker atenolol and placebo [24]. Hydrochlorothiazide + amiloride were shown to be more effective than atenolol for prevention of stroke and MI, as also found elsewhere [20, 24–26]. A report on blood pressure reduction in the treatment arms was also published [27] and recently updated [26]. But, although data on sodium, potassium, cholesterol, glucose, urate, urea and weight were collected for two years, there has been no report on how these parameters were affected. The continued use of hydrochlorothiazide makes these data highly relevant to current practice as does the data from the atenolol group. Beta-blockers block the action of catecholamines on portions of the sympathetic nervous system (those being the beta-adrenergic or *β*1 and *β*2 receptors) [28], in order to reduce heart rate, cardiac contractive force, and cardiac output [29]. Although they are no longer recommended by NICE as a first-line treatment option [1, 30], beta-blockers are known to have equal or greater effectiveness in the young compared with other anti-hypertensives [20, 25].

The objective of this work was to see how weight, potassium, sodium, cholesterol, glucose, urea and urate levels changed over time and whether the changes were different in the three arms. As noted, the results of the study in terms of blood pressure changes, mortality and cardio/cerebrovascular morbidity have already been published [24, 26, 27].

## Methods

### Data source and type

The data come from a Medical Research Council (UK) sponsored trial of anti-hypertensive medications in older adults which concluded in 1990 [24]. This was a randomized, placebo controlled, single blind trial (RCT) with two active arms (atenololand hydrochlorothiazide + amiloride) and a placebo arm. There were 4396 patients enrolled in total and they were followed for up to five years post-enrolment. Patients in diuretic arm received hydrochlorothiazide 50 mg plus amiloride 5 mg daily. However, due to metabolic disturbances observed after one year, all patients were transferred to a lower dose (hydrochlorothiazide 25 mg plus amiloride 2.5 mg). In the beta-blocker arm, patients received atenolol 50 mg once daily. If patients did not achieve target blood pressure after 12 weeks in this arm, the dose was increased (atenolol 100 mg); the calcium channel blocker nifedipine (doses up to 20 mg daily) and other supplementary drugs could be used if further control was needed [24]. The patients were seen by the research doctors and nurses at regular intervals in order to monitor their blood pressure and other parameters including weight, potassium, sodium, cholesterol, glucose, urea and urate levels. The latter set were measured at recruitment and end of years one and two of follow-up; unfortunately, there is no information available on the measurement protocol for these parameters.

### Missing data

Although the measurement schedule was fixed, not all patients have all the planned measurements owing to drop-out from the trial and because some patients may have died. The analyses here have been done on available data only but we conducted preliminary analyses to see whether those who later had missing data were different at baseline from those who did not.

### Statistical analysis

Changes over the first two years of the trial in potassium, sodium, glucose, weight and cholesterol were determined and compared between the randomised treatment arms regardless of adherence to treatment, i.e. according to the principle of Intention-To-Treat (ITT) analysis. The main analyses were based on fitting a Linear Mixed Models (LMM) to the data with time treated as a categorical variable and with an unstructured covariance structure; to study differences over time between groups, the model included group*time ‘interaction’ terms. Estimates of mean change within groups – and their Standard Errors (SE) - and differences between groups (hydrochlorothiazide + amiloride versus placebo, atenolol versus placebo and hydrochlorothiazide + amiloride versus atenolol) were extracted from these models. The advantage of this analysis approach over use of, say, paired and unpaired t-tests is that data from all patients can be used including from patients who had an incomplete data schedule. The variance components of the LMM models were set up to allow for the fact that within-group patient variability of most parameters tended to increase over time. The main focus of the analysis is the comparison of changes in the active treatment groups compared to each other and the placebo group.

## Results

### Background characteristics of patients

The 4396 patients enrolled in the trial were randomised to receive hydrochlorothiazide + amiloride (24.6 %), atenolol (25.1 %) or placebo (50.3 %). The median patient age (years) recorded during recruitment was 70 (ranging from 65 to 75) and 58.2 % (2,560) were female. At baseline, the mean glucose level amongst patients was 3.35 mmol/L; the average weight 70.16kgs; the mean potassium level 4.22 mmol/L; the mean sodium level 141.59 mmol/L; the mean cholesterol level 6.47 mmol/L, the mean urea 5.91 mmol/L and mean urate level 338.56 μmol/L. The baseline characteristics are compared between randomisation groups in Table 1; there are no important differences.

**Table 1:** Baseline characteristics of the study population (*n* = 4396)

Characteristics	Treatment group		
	Hydrochlorothiazide + amiloride	Atenolol	Placebo
*Number of patients enrolled*	1081 (24.6)	1102 (25.1)	2213 (50.3)
Male, *n* (%)	454 (42.0)	456 (41.4)	926 (41.8)
Age, years	70.4 ± 2.7	70.3 ± 2.8	70.3 ± 2.7
Weight, kg	70.1 ± 12.4	70.4 ± 12.5	70.0 ± 12.6
Serum potassium, mmol/L‡	4.2 ± 0.4 (*n* = 1046)	4.2 ± 0.4 (*n* = 1072)	4.2 ± 0.4 (*n* = 2160)
Serum sodium, mmol/L‡	141.6 ± 2.0 (*n* = 1055)	141.6 ± 2.0 (*n* = 1083)	141.6 ± 2.0 (*n* = 2173)
Glucose, mmol/L‡	3.4 ± 0.7 (*n* = 999)	3.4 ± 0.7 (*n* = 1025)	3.4 ± 0.7 (*n* = 2051)
Serum cholesterol, mmol/L‡	6.5 ± 1.3 (*n* = 1054)	6.5 ± 1.2 (*n* = 1075)	6.4 ± 1.2 (*n* = 2154)
Serum urea, mmol/L‡	6.0 ± 1.4 (*n* = 1036)	5.9 ± 1.4 (*n* = 1066)	5.9 ± 1.3 (*n* = 2128)
Serum urate, μmol/L‡	341.8 ± 70.5 (*n* = 1002)	336.8 ± 71.4 (*n* = 1030)	337.8 ± 70.5 (*n* = 2053)

‡Sample sizes for those with data on each measure are also shown

### Completed measurements per patient

Table 2 shows the number of measurements recorded per person at the three visits for various parameters. The majority of participants 3936 (89.5 %) had all 3 measurements taken for weight. Nearly three quarters of patients had all measurements recorded for sodium, potassium and urate. Almost two thirds of patients had all measurements for cholesterol, urea and glucose. The percentage of patients who died over the two years in the hydrochlorothiazide + amiloride arm was 12.4 %, in the atenolol arm 15.2 %, and in the placebo arm 14.2 %, which explains some of the missing data. Sixty-two patients (1.4 %) had died by end of year one.

**Table 2:** Number of measurements recorded per patient for each parameter (total patients = 4396)

Parameter	Number of measurements recorded *n* (%)			
	None	1	2	3
Weight, kg	5 (0.1)	0 (0.0)	455 (10.4)	3936 (89.5)
Serum potassium, mmol/L	21 (0.5)	494 (11.2)	636 (14.5)	3245 (73.8)
Serum sodium, mmol/L	15 (0.3)	489 (11.1)	598 (13.6)	3294 (74.9)
Glucose, mmol/L	45 (0.3)	561 (12.8)	913 (20.8)	2877 (65.5)
Serum cholesterol, mmol/L	22 (0.5)	490 (11.2)	652 (14.8)	3232 (65.7)
Serum urea, mmol/L	45 (1.0)	560 (12.7)	904 (20.6)	2887 (65.7)
Serum urate, μmol/L	18 (0.4)	512 (11.6)	737 (16.7)	3129 (71.2)

### Changes in outcome variables over time

Additional file 1: Table S1 shows the mean and SD of the measures for all treatment groups combined at each time point based on the available data for each visit; the numbers of patients vary between time points. These data show little change (less than 0.1 SD) in mean weight, glucose, cholesterol, or potassium over the first two years of the trial. The mean sodium decreased in the first year while urea and urate levels increased. Except for weight, the SDs of the parameters tended to increase over time.

### Comparisons between estimates of change over time between treatment groups

All available data for each parameter were included in the LMM statistical models. The models was used to estimate within-patient changes over time and to test whether these changes differed significantly between groups; these results are presented in Table 3. In considering results for each outcome, it is important to allow for any changes over time which might have occurred regardless of active treatment; hence changes in the placebo group are also shown.

**Table 3:** Mean (SE of mean) within-patient changes over time by treatment group

Changes	Potassium	Sodium	Glucose	Cholesterol	Weight	Urea	Urate
	Mean changes (SE)	Mean changes (SE)	Mean changes (SE)	Mean changes (SE)	Mean changes (SE)	Mean changes (SE)	Mean changes (SE)
Placebo							
Yr1 vs. Yr0	−0.00 (0.01)	−0.19 (0.05)**	−0.00 (0.02)	−0.03 (0.02)	−0.45 (0.08)**	0.08 (0.03)**	3.35 (1.09)**
Yr2 vs. Yr1	0.02 (0.1)**	−0.00 (0.05)	0.05 (0.02)**	0.07 (0.02)**	−0.23 (0.07)**	0.16 (0.03)**	3.89 (1.17)**
Hydrochlorothiazide + amiloride							
Yr1 vs. Yr0	−0.18 (0.01)*^,^ **	−1.64 (0.07)* ^,^**	0.03 (0.03)	0.13 (0.02)* ^*,*^ **	−0.91 (0.11)* ^*,*^ **	0.85 (0.04)* ^*,*^ **	56.45 (1.57)* ^*,*^ **
Yr2 vs. Yr1	0.04 (0.04)**	0.14 (0.08)	0.04 (0.03)	−0.01 (0.03)*	−0.26 (0.11)**	0.13 (0.05)**	−3.21 (1.67)*
Atenolol							
Yr1 vs. Yr0	0.07 (0.01)*^,^ **	−0.78 (0.07)* ^*,*^ **	−0.01 (0.03)	0.04 (0.02)*	0.05 (0.11)*	0.62 (0.04)* ^*,*^ **	43.00 (1.55)* ^*,*^ **
Yr2 vs. Yr1	−0.03 (0.01)*	−0.14 (0.08)	0.03 (0.03)	0.08 (0.03)**	−0.03 (0.11)	0.21 (0.05)**	4.81 (1.68)**

*Indicates *P* <0.05 and refers to test for difference in change in active treatment group compared to placebo group

**Indicates *P* <0.05 and refers to within-patient changes over time in each group

### Potassium

In the placebo arm, the change in the mean potassium level in year one compared to year zero was close to zero but there was a marginally significant increase in year 2 compared to year 1 of 0.02 mmol/L. At the end of year 1, patients in hydrochlorothiazide + amiloride arm had a statistically significant mean change in potassium level of −0.18 mmol/L compared to year zero, which was also significantly different from the (near zero) change in the placebo arm: (95 % CI for mean difference in changes: −0.21, −0.15). Furthermore, there was a significant change of 0.04 mmol/L in year two compared to year one in the hydrochlorothiazide + amiloride group, but this was not statistically significant compared to the placebo group change in the same period: (mean difference in changes 0.02 mmol/L, 95 % CI: −0.01, 0.07). In the atenolol arm, there was a statistically significant increase in the mean potassium level in year one compared to year zero of 0.07 mmol/L and a non-significant change of −0.03 mmol/L in year two compared to year one. The differences between atenolol arm and placebo arm in the changes in potassium in year 1 *vs.* year 0 and in year 2 *vs*. year 1 were statistically significant: mean difference in changes of 0.07 mmol/L, 95 % CI: (0.04, 0.11) and −0.5 mmol/L, 95 % CI: −0.08, −0.01) respectively. The differences between the hydrochlorothiazide + amiloride arm change (D) and atenolol arm change (B) were statistically significant: in year 1 *vs.* year 0, the mean difference (D-B) was −0.25 mmol/L, 95 % CI: (−0.29, −0.22), while for year 2 *vs.* year 1 changes, the mean difference (D-B) was 0.07 mmol/L, 95 % CI: (0.03, 0.11).

### Sodium

In the placebo group, the change in the mean sodium level in year one compared to year zero was −0.19 mmol/L and in year 2 compared to year 1 was close to zero. The former change was statistically significant (95 % CI: −0.09, −0.29). Compared to year zero, at the end of year 1, patients in hydrochlorothiazide + amiloride arm had a statistically significant mean change in sodium level of −1.64 mmol/L. This fall in the hydrochlorothiazide + amiloride group was significantly greater than in the placebo group (mean difference in changes −1.45 mmol/L, 95 % CI: −1.62, −1.29). There was a change of 0.14 mmol/L in year two compared to year one in the hydrochlorothiazide + amiloride group; this change was not statistically significant and not significantly greater compared to the placebo group (mean difference in changes 0.14 mmol/L, 95 % CI: −0.04, 0.33). In the atenolol arm, there was a statistically significant change in the mean sodium level in year one compared to year zero of −0.78 mmol/L and a non-significant change of −0.14 units in year two compared to year one. The difference in changes in sodium in atenolol group compared to placebo group was statistically significant (mean difference in changes −0.59 mmol/L, 95 % CI: −0.76, −0.42) for year 1 *vs.* year 0 but not significant for year 2 *vs*. year 1 (mean difference in changes −0.13 mmol/L, 95 % CI: −0.32, −0.06). Also, the difference between the hydrochlorothiazide + amiloride arm and atenolol arm changes were statistically significant but in different directions in the two periods: in year 1 *vs.* year 0, the mean difference (D-B) was −0.87 mmol/L, 95 % CI: (−1.07, −0.67) while in year 2 *vs.* year 1, it was 0.27 mmol/L, 95 % CI: (0.05, 0.50).

### Glucose

The change in the mean glucose level in year one compared to year zero in the placebo arm was close to zero and 0.05 mmol/L in year 2 compared to year 1. The latter change was statistically significant (95 % CI: 0.01, 0.09). Patients in the hydrochlorothiazide + amiloride arm had a non-significant mean change in glucose level of 0.03 mmol/L in year one compared to year zero and 0.04 mmol/L in year two compared to year one. The differences in the changes in glucose in year 1 *vs.* year 0 and year 2 *vs*. year 1 between hydrochlorothiazide + amiloride arm and placebo arm were not statistically significant (. mean difference in changes 0.03 mmol/L, 95 % CI: −0.04, 0.09) and −0.01 mmol/L, 95 % CI: −0.08, 0.06) respectively. In the atenolol arm, there was a non-significant change in the mean glucose level in year one compared to year zero of −0.01 mmol/L and 0.03 mmol/L in year two compared to year one. The differences in the changes in glucose in year 1 *vs.* year 0 and year 2 *vs*. year 1 between atenolol arm and placebo arm were not statistically significant i.e. mean difference in changes of −0.01 mmol/L, 95 % CI: −0.07, 0.05) and (−0.02 mmol/L), (95 % CI) (−0.08, 0.05) respectively. Likewise the difference between the hydrochlorothiazide + amiloride arm and atenolol arm changes (D-B) were not statistically significant: 0.04 mmol/L, 95 % CI: (−0.04, 0.11) in year 1 vs. year 0, and 0.01 mmol/L, 95 % CI: (−0.07, 0.09) in year 2 *vs*. year 1.

### Cholesterol

In the placebo arm, the mean change in cholesterol level in year one compared to year zero was −0.03 mmol/L and in year 2 compared to year 1 was 0.07 mmol/L. The latter change was statistically significant (95 % CI: 0.03, 0.11). Compared to year zero, at the end of year 1, patients in hydrochlorothiazide + amiloride arm had a statistically significant mean change in cholesterol level of 0.13 mmol/L. This change was significantly different compared to the placebo group change (mean difference in changes 0.16 mmol/L, 95 % CI: 0.10, 0.22). There was a non-significant change of −0.01 mmol/L in year two compared to year one in the hydrochlorothiazide + amiloride group, but this was significantly different from the placebo group whose cholesterol has increased in this period (mean difference in changes −0.08 mmol/L, 95 % CI: −0.15, −0.02). In the atenolol arm, there was a non-significant change in the mean cholesterol level in year one compared to year zero of 0.04 mmol/L and a statistically significant change of 0.08 mmol/L in year two compared to year one. The difference in changes in cholesterol levels between the atenolol group and the placebo group was statistically significant in year 1 *vs.* year 0 (mean difference in changes 0.07 mmol/L, 95 % CI: 0.02, 0.13) but not significant in year 2 *vs*. year 1 (mean difference in changes 0.01 mmol/L, 95 % CI: −0.05, 0.07). Also, the difference between the hydrochlorothiazide + amiloride arm and atenolol arm changes were statistically significant but in different directions: in year 1 *vs.* year 0, the mean difference (D-B) was 0.08 mmol/L, 95 % CI: (0.02, 0.15) while in year 2 *vs.* year 1, it was −0.09 mmol/L, 95 % CI: (−0.17, −0.02).

### Weight

In the placebo arm, there were statistically significant change in mean weight in year one compared to year zero −0.45 kgs 95 % CI (−0.29, −0.60) and in year 2 compared to year 1: −0.23 kgs 95 % CI: (−0.38, −0.09). Patients in hydrochlorothiazide + amiloride arm had a statistically significant mean change in weight of −0.91 kgs in year one compared to year zero and −0.26 kgs in year two compared to year one. The differences between hydrochlorothiazide + amiloride arm and placebo arm in the changes in weight in year 1 *vs.* year 0 was statistically significant (mean difference in changes of −0.46 kgs, 95 % CI: −0.73, −0.20) but not in year 2 *vs*. year 1, (mean difference in changes of −0.03 kgs, 95 % CI: −0.29, 0.23). In the atenolol arm, there were non-significant changes in mean weight in year one compared to year zero of 0.05 kgs and of −0.03 kgs in year two compared to year one. The differences in the changes in weight between atenolol arm and placebo arm in year 1 *vs.* year 0 was statistically significant (mean difference in changes of 0.50 kgs, (95 % CI: 0.23, 0.76) but not significant between year 2 *vs*. year 1 (mean difference in changes of 0.20 kgs, 95 % CI: −0.06, 0.46). The difference between the hydrochlorothiazide + amiloride arm and atenolol arm changes in weight was statistically significant in the first period - mean difference (D-B) of −0.96 kgs, 95 % CI: (−1.27, 0.65) but not in the second - mean difference (D-B) of −0.23 kgs, 95 % CI: (−0.57, 0.10).

### Urea

In the placebo arm, there were statistically significant mean changes in urea in year one compared to year zero - 0.08 mmol/L (95 % CI: 0.03, 0.14) - and in year 2 compared to year 1 – 0.16 mmol/L (95 % CI: 0.10, 0.22). Patients in the hydrochlorothiazide + amiloride arm had a statistically significant mean change in urea of 0.85 mmol/L (95 % CI: 0.78, 0.93) in year one compared to year zero and of 0.13 mmol/L (95 % CI: 0.03, 0.22) in year two compared to year one respectively. The differences in the changes in urea between hydrochlorothiazide + amiloride arm and placebo arm in year 1 *vs.* year 0 was statistically significant (mean difference in changes of 0.77 mmol/L, (95 % CI: 0.68, 0.87) but not statistically significant in year 2 *vs*. year 1 (mean difference in changes of −0.03 mmol/L, 95 % CI: −0.15, 0.08). In the atenolol arm, there were statistically significant changes in the mean urea in year one compared to year zero of 0.62 mmol/L (95 % CI: 0.54, 0.70) and 0.21 mmol/L (95 % CI: 0.11, 0.302) in year two compared to year one. The differences in the changes in urea between atenolol arm and placebo arm in year 1 *vs.* year 0 was statistically significant (mean difference in changes of 0.54 mmol/L, 95 % CI: 0.44, 0.63) but non-significant between year 2 *vs*. year 1 (. mean difference in changes of 0.05 mmol/L, 95 % CI: −0.07, 0.16). The difference between the hydrochlorothiazide + amiloride arm and atenolol arm changes was statistically significant for year 1 *vs.* year 0 - mean difference (D-B) was 0.24 mmol/L, 95 % CI: (0.13, 0.35 - but not in year 2 *vs.* year 1 - mean difference (D-B) was −0.08 mmol/L, 95 % CI: (−0.22, 0.05).

### Urate

In the placebo group, the changes in urate in year one compared to year zero was 3.35 μmol/L (95 % CI: 1.21, 5.49) and in year 2 compared to year 1 was 3.89 μmol/L (95 % CI: 1.59, 6.19). Patients in the hydrochlorothiazide + amiloride arm had a statistically significant mean change in urate of 56.45 μmol/L (95 % CI: 53.37, 59.52) in year one compared to year zero and a non-significant change of −3.21 μmol/L (95 % CI: −6.48, 0.06) in year two compared to year one. The differences in the changes in urate between hydrochlorothiazide + amiloride arm and placebo arm in year 1 *vs.* year 0 and year 2 *vs*. year 1 were statistically significant (mean difference in changes of 53.10 μmol/L, 95 % CI: 49.35, 56.85 and of −7.10 μmol/L, 95 % CI: −11.09, −3.10 respectively). In the atenolol arm, there was a statistically significant change in the mean urate in year one compared to year zero of 43.00 μmol/l (95 % CI: 39.96, 46.04) and 4.81 μmol/L (95 % CI: 1.53, 8.10) in year two compared to year one. The difference in the changes in urate between atenolol arm and placebo arm in year 1 *vs.* year 0 was statistically significant (mean difference in changes 39.65 μmol/L, 95 % CI: 35.93, 43.37) but not significant between year 2 *vs*. year 1 (mean difference in changes of 0.93 μmol/L, 95 % CI: −3.08, 4.93). The difference between the hydrochlorothiazide + amiloride arm and atenolol arm changes was statistically significant in both periods but not in the same direction: in year 1 vs. year 0, the mean difference (D-B) was 13.43 μmol/L, 95 % CI: (9.10, 17.76), while in year 2 vs. year 1, the mean difference (D-B) was −8.02 μmol/L, 95 % CI: (−12.75, −3.30).

## Discussion

The conduct of this trial has been described elsewhere: no serious problems in terms of validity have been reported [24, 26, 27]. As already noted, hydrochlorothiazide + amiloride were shown to be more effective than atenolol for prevention of stroke and MI [24]. Although the hydrochlorothiazide + amiloride arm showed the greatest drop-off in BP in the initial stage of the trial, both active treatment groups reached similar average levels after 2 years of follow-up [26]. Therefore, it was of interest to see whether there was evidence that other metabolic parameters - which could play a part in patient morbidity and mortality - were affected by these treatments.

Given complete data arising from a randomised controlled trial, ITT comparisons between randomised groups allow us to estimate the causal effect of the ’intention to treat’ with a drug. However true efficacy can be underestimated by ITT comparisons when there is non-adherence or treatment cross-over as the trial progresses. The ability to produce unbiased evidence may also be compromised when there is missing data. However, this issue is less problematic in the first year of the trial: data was 83–90 % complete for all parameters except glucose (77 %) at end of year one.

In the first year of the trial, patients in diuretic arm received hydrochlorothiazide 25 mg or 50 mg plus amiloride 2.5 mg or 5 mg daily [24]. Due to metabolic disturbances observed among patients who were using highest dose, all patients transferred to a lower dose (hydrochlorothiazide 25 mg plus amiloride 2.5 mg). Some of the observed differences between year 1 and year 2 changes among patients randomised in diuretic arm might be explained by changes in dosage. Also we would expect treatment supplementation may have been more pronounced in the second year compared to the first, thus blurring the contrasts between treatment effects. These considerations suggest more emphasis is placed on the year one results, which we now discuss in terms of changes relative to placebo arm changes.

In the first year, hydrochlorothiazide + amiloride treatments appeared to reduce sodium, weight *and* potassium compared to placebo. The reduction in sodium that might be attributed to the treatment, i.e. the difference between hydrochlorothiazide + amiloride and placebo arm changes, was substantial – on average, equivalent to 0.72 of baseline SD (bSD). The beta-blocker, atenolol, also reduced sodium compared to placebo but to a lesser extent (0.29 bSD), and had little effect on weight i.e. weight gain (0.04 bSD). This combination of findings supports the previous research which showed diuretic treatments being associated with weight losses rather than the beta blockers which are known to be associated with weight gains [10, 11, 31, 32]. The decrease in potassium attributable to the hydrochlorothiazide + amiloride treatments was non-trivial – equivalent to 0.46 bSD, while mean potassium levels were increased by 0.19 bSD in the atenolol group. The changes in serum potassium in hydrochlorothiazide + amiloride group were in line with findings from previous studies [33, 34]. Potassium-sparing diuretics, despite not fully - are known to correct the fall in serum potassium by increasing serum potassium concentrations [35]. The impacts of beta blocker on potassium match those observed in earlier studies and are not of clinical importance [13, 36].

From the present data, it appeared that neither regimen affect glucose levels in the first year; this is surprising given a report elsewhere of withdrawal rates of 6.9, 5.8 and 2.7 per 1000 person-years -which we estimate as 4.0 %, 3.4 % and 1.5 % of patients - in hydrochlorothiazide + amiloride, atenolol and placebo arms respectively for “impaired glucose tolerance” [24]. In our data, no patient in the hydrochlorothiazide + amiloride arm and only one in the atenolol arm had a glucose level higher than 7umol/L compared to eight in the placebo arm. This incompatibility with results from the original report suggests that our data may be incomplete and therefore our results should be downplayed.

Cholesterol levels increased significantly over year one in the hydrochlorothiazide + amiloride arm; measured against the change in the placebo group, the increase attributable to treatment was of the order of 0.13 bSD; the atenolol arm also showed a significant increase compared to placebo which again was rather small (0.06 bSD). These findings differ from those obtained by Lakshaman et al. which showed none of the drugs had a long-term adverse effects on plasma lipids and lipoprotein profiles in men with hypertension [37]. The increase in cholesterol levels in hydrochlorothiazide + amiloride arm is in accord with previous studies which indicated relative increase in cholesterol level which varied with dose and/or race [38, 39].

Both active treatments arms showed large increases in mean urea and mean urate levels over year one, but the increases for the hydrochlorothiazide + amiloride group were significantly greater than in the atenolol group. For urea, the increases were equivalent to 0.56 bSD and 0.39 bSD respectively, while for urate they were 0.75 bSD and 0.56 bSD. Other studies have shown consistent finding that diuretic and beta blocker treatments gives similar changes in kidney function [36, 39]. Increases in urate levels associated with treatment with diuretics (but not potassium-sparing diuretics) and beta blockers have been reported previously [8, 18, 36, 40] and so results here are not unexpected. Bengtsson has recommended monitoring of serum uric acid levels after treatments with diuretics [40]. However, in recent times, there has been new debate [41–43], as to its significance for both hypertension and cardiovascular disease: “an innocent bystander” or a “central player”? Commenting on work by Viazzi et al., which found that increased serum uric acid (sUA) levels at baseline blunted the antihypertensive impact 1.5 years later of lifestyle changes in children, Bavish concluded that sUA is emerging “as a key factor modulating hypertension”. Elsewhere [44], in an observational study based on the UK CPR Database, hypertensive patients over 65 years with stable BP medication who were also prescribed allopurinol showed a small reduction in SBP and DBP over time compared to comparable patients who were not, again suggesting a causal role for sUA, although baseline sUA did not predict in this study. In view of this and other evidence, the increase in sUA in the hydrochlorothiazide + amiloride and atenolol arms in this study are notable: perhaps BP reduction might have been greater has there not been concomitant increases in urate.

The key strengths of this study are its large sample size, relative long follow-up and the use of sophisticated statistical methods in analysis (LMM). This method of statistical analysis uses all data from all patients including those with an incomplete data schedule. Furthermore, the analysis involved estimation of changes within and between treatment groups over time. Although parameters may not have changed sufficiently to warrant withdrawal from the study for most patients, for scientific reasons, it is important to understand the degree of average change in the group, including when there is little change. Indeed, results from large, well-powered studies, which show little change, are important for demonstrating safety.

Despite being a multicentre study whereby measurements were not performed in the same core laboratory – the results of this study are not likely to be affected by inter laboratory differences. The results which compare the same patients at different time points (within-patient comparisons) and comparisons between treatment groups are not likely to be affected by inter laboratory differences in technique as we presume each clinic used one laboratory and the same laboratory was used for a given patient. Furthermore, randomisation to treatment groups was in stratified blocks within each sex and clinic.

Our ‘intention to treat’ analysis followed the approach of the original mortality study by comparing outcomes by intended treatment, despite the treatment changes within the treatment period. Such analyses are acknowledged to address important pragmatic questions [45].

It might be argued that patient aged 65–75 are healthier today than 30 years ago but the evidence suggests that deterioration is merely postponed: “indices of heath that used to prevail at age 70 now prevail at age 80” [46]. We believe that these results remain relevant to patients who present with systolic pressures of 160 mmHg or more and who are prescribed similar drugs. As noted in the Introduction, the US 2014 recommendations for first line treatments continue to include thiazide type diuretics as well as beta blockers [5].

## Conclusion

This study has shown that hydrochlorothiazide + amiloride and atenolol treatments increase cholesterol, urea and urate levels in a short period of time. Both active treatments arms showed a significant decrease in sodium levels over the first year of the trial. , No differences were found in glucose levels among patients randomised in hydrochlorothiazide + amiloride and atenolol arms when compared to placebo arm.

## Additional file

Additional file 1: Table S1. Mean (SD) of outcome variables over time (all groups combined). (DOCX 12 kb)

## Abbreviations

ACEs: Angiotensin converting enzyme inhibitors
ARBs: Angiotensin receptor blockers
bSD: Baseline standard deviation
CCBs: Calcium channel blockers
CI: Confidence Interval
ITT: Intention-To-Treat
LMM: Linear mixed model
MI: Myocardial Infarction
MRC: Medical Research Council
SD: Standard deviation
SE: Standard error
UK: United Kingdom
US: United States
