# Supplementary material for: Changes in selected metabolic parameters in patients over 65 receiving hydrochlorothiazide plus amiloride, atenolol or placebo in the MRC elderly trial
Source: BMC Cardiovasc Disord. 2016 Oct 4;16:188. doi: 10.1186/s12872-016-0368-2 (PMC5050956; doi:10.1186/s12872-016-0368-2)
Supplement: Additional file 1: Table S1. — Mean (SD) of outcome variables over time (all groups combined). (DOCX 12 kb) [file 12872_2016_368_MOESM1_ESM.docx]

**Additional file 1: Table S1: Mean (SD) of outcome variables over time (all groups combined)**

| **Parameter** | **Visits**  *mean* ±*SD* | | |
| --- | --- | --- | --- |
|  | **Baseline** | **First year** | **Second year** |
| Weight, kg | 70.16±12.50 | 69.81±12.42 | 69.74±12.45 |
| Serum potassium, mmol/L | 4.22± 0.37 | 4.19± 0.41 | 4.20± 0.41 |
| Serum sodium, mmol/L | 141.59± 2.01 | 140.91± 2.46 | 140.91± 2.49 |
| Glucose, mmol/L | 3.35± 0.68 | 3.36± 0.70 | 3.40± 0.73 |
| Serum cholesterol, mmol/L | 6.47±1.24 | 6.51±1.26 | 6.57±1.27 |
| Serum urea, mmol/L | 5.91±1.37 | 6.32±1.64 | 6.47±1.80 |
| Serum urate, µmol/L | 338.56±70.74 | 365.00±81.41 | 367.74±81.64 |
